# Investigating the Roles of Listeria monocytogenes Peroxidases in Growth and Virulence

**DOI:** 10.1128/spectrum.00440-21

**Published:** 2021-07-21

**Authors:** Monica R. Cesinger, Nicole H. Schwardt, Cortney R. Halsey, Maureen K. Thomason, Michelle L. Reniere

**Affiliations:** a Department of Microbiology, University of Washington grid.34477.33grid.471394.c School of Medicine, Seattle, Washington, USA; University of Guelph

**Keywords:** heme, oxidative stress, peroxide, redox signaling, virulence

## Abstract

Bacteria have necessarily evolved a protective arsenal of proteins to contend with peroxides and other reactive oxygen species generated in aerobic environments. Listeria monocytogenes encounters an onslaught of peroxide both in the environment and during infection of the mammalian host, where it is the causative agent of the foodborne illness listeriosis. Despite the importance of peroxide for the immune response to bacterial infection, the strategy by which L. monocytogenes protects against peroxide toxicity has yet to be illuminated. Here, we investigated the expression and essentiality of all the peroxidase-encoding genes during L. monocytogenes growth *in vitro* and during infection of murine cells in tissue culture. We found that *chdC* and *kat* were required for aerobic growth *in vitro*, and *fri* and *ahpA* were each required for L. monocytogenes to survive acute peroxide stress. Despite increased expression of *fri, ahpA*, and *kat* during infection of macrophages, only *fri* proved necessary for cytosolic growth. In contrast, the proteins encoded by *lmo0367*, *lmo0983*, *tpx*, *lmo1609*, and *ohrA* were dispensable for aerobic growth, acute peroxide detoxification, and infection. Together, our results provide insight into the multifaceted L. monocytogenes peroxide detoxification strategy and demonstrate that L. monocytogenes encodes a functionally diverse set of peroxidase enzymes.

**IMPORTANCE** Listeria monocytogenes is a facultative intracellular pathogen and the causative agent of the foodborne illness listeriosis. L. monocytogenes must contend with reactive oxygen species generated extracellularly during aerobic growth and intracellularly by the host immune system. However, the mechanisms by which L. monocytogenes defends against peroxide toxicity have not yet been defined. Here, we investigated the roles of each of the peroxidase-encoding genes in L. monocytogenes growth, peroxide stress response, and virulence in mammalian cells.

## INTRODUCTION

Bacteria that replicate in aerobic environments must contend with reactive oxygen species (ROS) such as superoxide (O_2_^−^), hydroxyl radicals (HO·), and hydrogen peroxide (H_2_O_2_). These ROS result from the incomplete reduction of molecular oxygen and are both produced by the bacteria (endogenous) and encountered extracellularly (exogenous). Accumulation of ROS leads to DNA damage and mutagenesis, protein oxidation, and destruction of iron-sulfur clusters and other iron-containing proteins (1). In light of these deleterious effects, it is perhaps unsurprising that the mammalian immune system uses ROS to defend against invading pathogens via the phagocytic respiratory burst, a period of increased oxygen consumption observed during phagocytosis due to the activity of NADPH oxidase (2). NADPH oxidase generates ROS in the phagosome and is critical to the innate immune response, as evidenced by the increased susceptibility of patients with chronic granulomatous disease (CGD) to severe infections. CGD is an inherited immunodeficiency caused by deletions or mutations in the genes encoding NADPH oxidase (3). Without this immune defense mechanism, CGD patients suffer from invasive and recurrent infections (4).

Bacteria employ many defense mechanisms to defend themselves against ROS-mediated oxidative stress, the most impactful being the production of scavenging enzymes (5). Superoxide dismutase detoxifies superoxide, generating molecular oxygen and hydrogen peroxide, which is then efficiently scavenged by peroxidases and catalases. Peroxidases are enzymes that reduce hydrogen peroxide and are the primary scavengers at low concentrations of peroxide. At high concentrations of peroxide, when peroxidases become saturated, catalases carry out disproportionate of peroxide and become the dominant scavengers (6). Bacteria typically produce multiple functionally redundant enzymes to protect against peroxides. In fact, peroxide toxicity is not detected in the model organism Escherichia coli unless three peroxide scavenging enzymes are deleted simultaneously (*ahpCF*, *katG*, and *katE*) (7).

While the mechanisms of peroxide detoxification have been studied for decades in the model organisms E. coli and Bacillus subtilis (5, 6, 8), the mechanisms by which Listeria monocytogenes defends against ROS are less clear. L. monocytogenes is a facultative foodborne pathogen that invades host cells, replicates in the cytosol, and spreads to neighboring cells using actin-based motility (9). The virulence factors employed by L. monocytogenes to successfully infect a host are all transcriptionally regulated by the master virulence regulator PrfA, which is itself redox regulated (10). In addition to virulence factors, PrfA regulates genes that enhance L. monocytogenes resistance to peroxide (11), further indicating that peroxide stress is relevant during infection. We became particularly interested in the peroxide detoxification strategy employed by L. monocytogenes during infection when our previous work revealed that the single catalase (encoded by *kat*) produced by the bacterium is required for aerobic growth, but dispensable during infection (12). These results led to the hypothesis that L. monocytogenes produces other redundant peroxidases in order to survive the respiratory burst of the macrophage phagosome. Here, we evaluated the expression and essentiality of all peroxidase-encoding genes during L. monocytogenes growth *in vitro* and during infection of murine cells in tissue culture.

## RESULTS

### L. monocytogenes encodes 9 predicted peroxidases.

To identify all the proteins with predicted peroxidase activity encoded by L. monocytogenes (Table 1), we performed an *in silico* analysis. Specifically, the RedoxiBase database includes the peroxidases produced by Bacillus subtilis, and each of these was used as a query protein sequence to identify homologous proteins produced by L. monocytogenes 10403S (13). According to the RedoxiBase database, peroxidase proteins belong to two families: heme peroxidases and nonheme peroxidases (13). Of the heme peroxidases, L. monocytogenes encodes a heme-dependent catalase (Kat), a coproheme decarboxylase (ChdC, formerly HemQ), and a DyP-type peroxidase (Lmo0367). The nonheme peroxidases include the peroxiredoxin family (Thiol Prx superfamily), which is characterized by highly conserved peroxidatic cysteine residues (6). In this family, L. monocytogenes encodes Lmo0983, AhpA (formerly AhpC or Prx), Tpx, Lmo1609, and OhrA. Finally, Fri is a nonheme bacterial ferritin classified as an oxidoreductase by the RedoxiBase database (13).

**TABLE 1:** Predicted peroxidases encoded by L. monocytogenes

Gene	Name(s)	Predicted function	Reference(s)
*lmo0367*		Heme-dependent DyP-type peroxidase	
*lmo0943*	*fri, dps*	Bacterioferritin, oxidoreductase	14, 15
*lmo0983*		Glutathione peroxidase	
*lmo1583*	*tpx*	Thiol peroxidase	
*lmo1604*	*ahpA*	2-Cys peroxiredoxin	24, 25
*lmo1609*		Thioredoxin	
*lmo2113*	*chdC, hemQ*	Putative heme peroxidase, involved in heme biosynthesis	22
*lmo2199*	*ohrA*	Organic hydroperoxidase	16
*lmo2785*	*kat*	Heme-dependent catalase	12

Prior research on the role of L. monocytogenes peroxidases is limited. The best studied is the ferritin protein Fri (formerly Dps), which was shown to be important for the acute peroxide stress response, long-term stationary-phase survival, and adaptation to shifting growth conditions (14, 15). A L. monocytogenes strain lacking *ohrA* is more sensitive to a variety of oxidative stressors and is attenuated during infection (16). The most dramatic phenotype associated with a L. monocytogenes peroxidase mutant is exhibited by strains lacking *kat*, which replicate aerobically to mid-log phase at the same rate as the wild type (wt) but then succumb to endogenously produced peroxide toxicity (12). However, *kat* is not required for intracellular replication in macrophages or for virulence in a murine model of infection, and catalase-deficient strains have been isolated from infected humans (12, 17, 18). Based on the important role of peroxide in the innate immune response and the limited research on L. monocytogenes peroxidases, we sought a more holistic picture of the role of peroxidases in aerobic growth and virulence.

### Expression of peroxidase-encoding genes.

**FIG 1:**
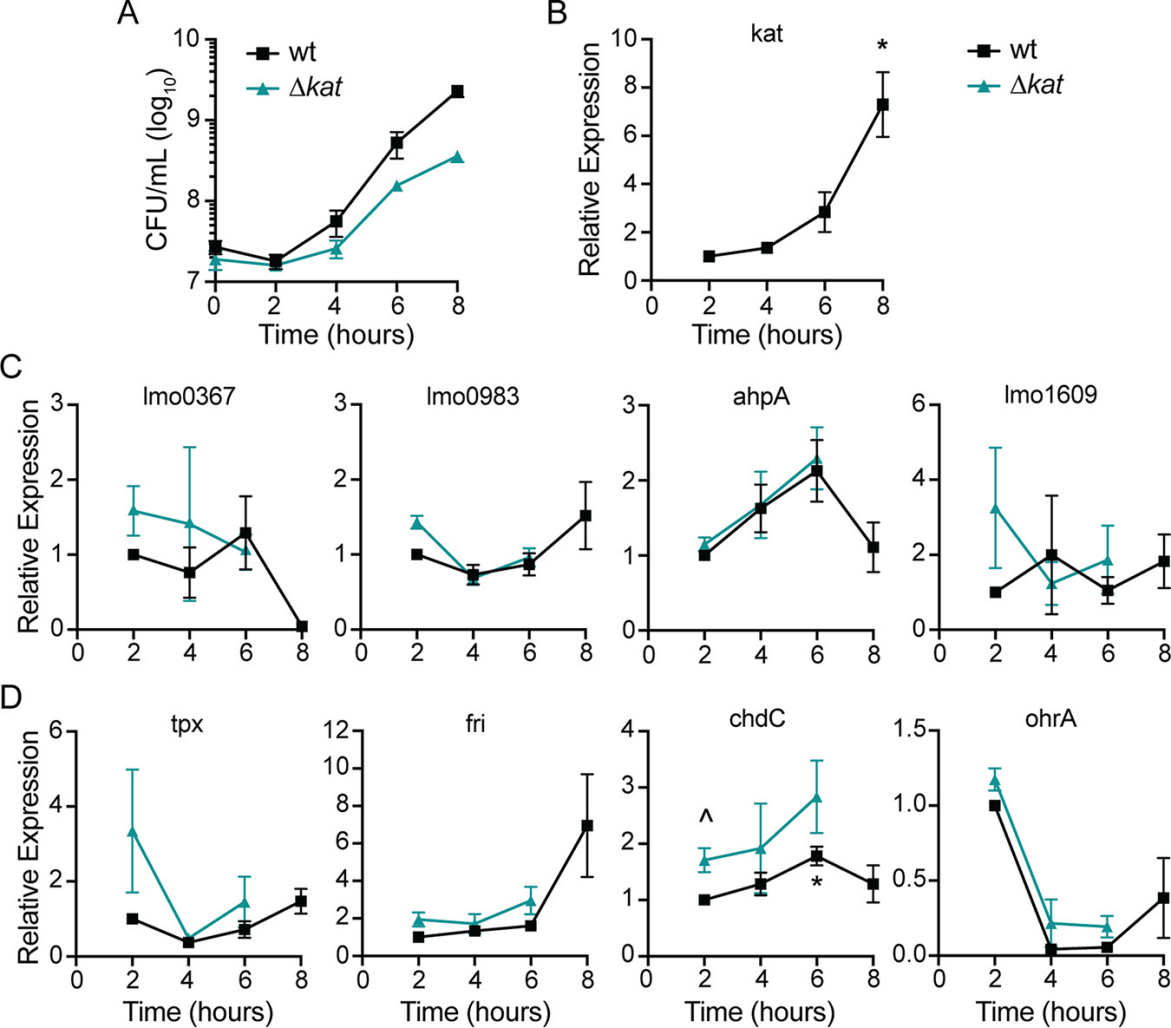
Expression of genes encoding putative peroxidases during aerobic growth. (A) Aerobic growth of wt and Δ*kat* strains in shaking flasks was measured by plating for CFU and incubating the plates anaerobically. Data are means and standard errors of the means (SEM) for three biological replicates. (B to D) Relative expression of putative peroxidase-encoding genes over time in both wt and Δ*kat* strains, grown as described for panel A. Expression was normalized to wt expression at 2 h. Data are means and SEM for at least three biological replicates. *P* values were calculated using a heteroscedastic Student's *t* test. *, *P* < 0.05 for expression compared to the wt at 2 h; ^, *P* < 0.05 for expression in the Δ*kat* mutant compared to the wt at that time point.

To investigate which peroxidase enzymes may be important during L. monocytogenes aerobic growth, we first evaluated gene expression in rich medium by quantitative reverse transcriptase PCR (qPCR). In these experiments, bacteria were grown in tryptic soy broth (TSB) which lacks glutathione and heme, both of which influence the redox environment of bacterial cultures. Expression of each putative peroxidase-encoding gene was evaluated in wt and Δ*kat* strains and expression of transcripts was normalized to wt values at 2 h after inoculation into shaking flasks. The Δ*kat* mutant replicated to mid-log phase but stopped growing and began to die 8 h postinoculation (Fig. 1A) (12). This requirement for Kat activity in early stationary phase corresponds to the peak expression of *kat* in wt L. monocytogenes (Fig. 1B). Accordingly, we found that gene expression could not be reliably measured in a Δ*kat* mutant 8 h postinoculation due to bacterial death. Analyzing transcript abundance of the other peroxidase-encoding genes revealed that *lmo0367*, *lmo0983*, *ahpA*, and *lmo1609* transcripts did not increase appreciably over time in the wt or Δ*kat* strains (Fig. 1C). In contrast, *tpx* expression was elevated early in L. monocytogenes lacking *kat* compared to the wt, although this was not statistically significant (Fig. 1D) (*P* =  0.2). The gene encoding Fri was increased in wt L. monocytogenes 8 h postinoculation, similarly to *kat* expression. Expression of *chdC* exhibited a small but statistically significant increase initially in L. monocytogenes lacking *kat* compared to the wt and was significantly increased in the wt after 6 h of aerobic growth (Fig. 1D). The gene encoding OhrA exhibited maximal expression 2 h postinoculation and decreased dramatically over time in both strains. Taken together, these data demonstrated that *kat* and *fri* share similar expression dynamics during aerobic growth.

**FIG 2:**
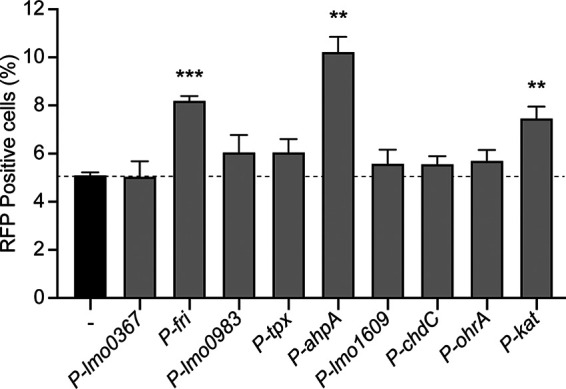
Intracellular expression of peroxidase-encoding genes. J774 macrophages were infected with each reporter strain, which expressed *rfp* from the indicated promoter and constitutive *gfp*. Cells were infected for 6 h and then analyzed by flow cytometry. The dotted line indicates background RFP fluorescence. Data are means and SEM for three biological replicates. *P* values were calculated using a heteroscedastic Student's *t* test comparing each mutant to the wt. **, *P* < 0.01; ***, *P* < 0.001.

We next measured expression of these peroxidase-encoding genes during infection using fluorescent transcriptional reporters. Strains were engineered to express *rfp* from the native promoter of each peroxidase-encoding gene, and *gfp* was expressed constitutively. J774 macrophages were infected for 1 h before gentamicin was added to the medium to eliminate extracellular bacterial growth. Flow cytometry was performed 6 h postinfection and infected cells were identified by green fluorescent protein (GFP) fluorescence compared to an uninfected control. Cells infected with the reporter strains for expression of *fri*, *ahpA*, and *kat* exhibited significantly increased red fluorescent protein (RFP) production compared to the background (Fig. 2). These results suggested that only *fri*, *ahpA*, and *kat* are significantly expressed during macrophage infection and therefore might have roles in virulence.

### Growth of peroxidase mutants in broth.

Previous work demonstrated that L. monocytogenes requires *kat* to detoxify endogenously produced peroxide during aerobic growth (12). Here, we sought to test if additional peroxidases are important for aerobic growth. For this analysis, each potential peroxidase-encoding gene was deleted by allelic exchange while the strain was growing anaerobically to prevent oxygen-mediated toxicity. Based on the redundant roles of Kat and Ahp in other bacteria (7, 19), we also generated a Δ*kat* Δ*ahpA* double mutant. Anaerobic bacterial overnight cultures were diluted into rich medium and grown aerobically in shaking flasks. Serial dilutions were performed to enumerate CFU over time, and the plates were incubated anaerobically to promote growth of potentially oxygen-sensitive strains.

**FIG 3:**
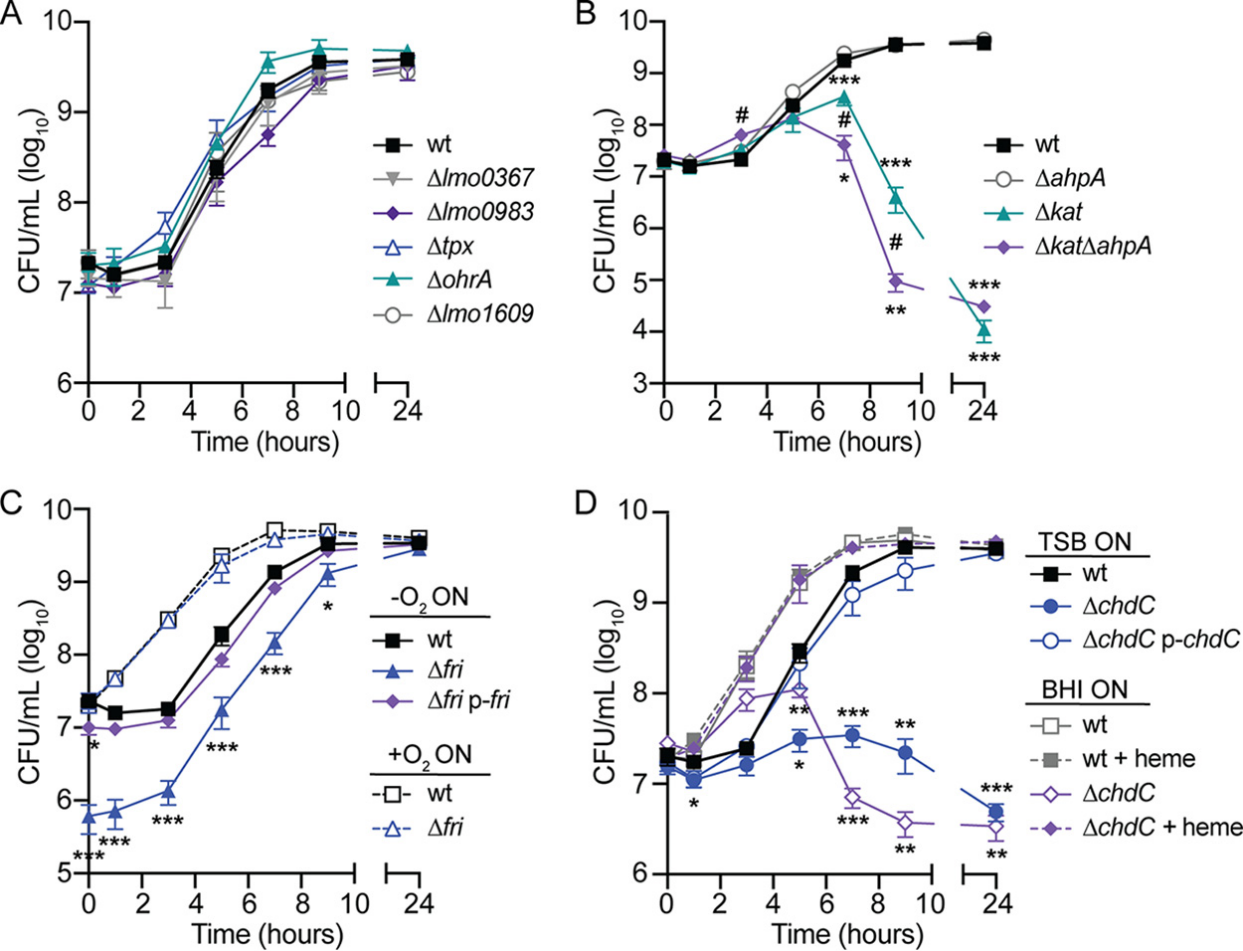
Aerobic growth of peroxidase mutants. (A) Growth curves of strains that replicate at the same rate as the wt (*P* > 0.05 at each time point). (B) Growth curves comparing Δ*ahpA*, Δ*kat*, and Δ*kat ΔahpA* mutants. ^#^, *P* < 0.05 between the Δ*kat* and Δ*kat ΔahpA* strains. (C) Growth curves of strains grown anaerobically (solid lines) or aerobically (dotted lines) overnight before back-diluting into shaking flasks. (D) Strains were grown anaerobically overnight in TSB or BHI and then diluted into TSB with or without exogenous heme (5 μM). In all panels, data are means and SEM for three biological replicates. *P* values were calculated using a heteroscedastic Student's *t* test comparing each mutant to the wt grown under the same conditions. *, *P* < 0.05; **, *P* < 0.01; ***, *P* < 0.001.

Growth analyses revealed that several peroxidase-encoding genes were dispensable for aerobic replication, including *lmo0367*, *lmo0983*, *tpx*, *ohrA*, *lmo1609*, and *ahpA* (Fig. 3A and B). In contrast, *kat* was required for aerobic growth, and strains lacking this gene began to die upon entry into stationary phase (Fig. 3B), as previously reported (12). The Δ*kat* Δ*ahpA* double mutant was even more sensitive to oxygen, as we observed a significant attenuation in survival at 7 and 9 h postinoculation compared to the single mutant lacking *kat* (Fig. 3B). These data indicate that AhpA is functional and important for aerobic replication in the absence of catalase.

Interestingly, we observed a dramatic phenotype for the L. monocytogenes strain lacking *fri* (Fig. 3C). Despite normalizing the anaerobic overnight cultures by optical density, we consistently observed a 1- to 2-log defect in Δ*fri* CFU at the initial time point, and this defect continued throughout growth until stationary phase (Fig. 2C). This aerobic growth defect could be complemented by providing a copy of *fri* in *trans* using the integrative plasmid pPL2 (p-*fri*) (20). Previous work suggested that *fri* is important for L. monocytogenes adaptation to stress conditions, including nutritional stress and temperature shifts (15). We therefore hypothesized that the Δ*fri* mutant was unable to rapidly adapt from hypoxia to aerobic growth. In support of this hypothesis, we found that Δ*fri* incubated aerobically overnight grew similarly to the wt after dilution into shaking flasks (Fig. 3C, dashed lines). Consequently, the Δ*fri* mutant was incubated overnight aerobically for all subsequent experiments.

Finally, we observed the Δ*chdC* mutant did not replicate when diluted into shaking flasks, and this growth defect was genetically complemented by expressing *chdC* in *trans* (p-*chdC*) (Fig. 3D). This finding is in agreement with published work reporting that *chdC* is essential in L. monocytogenes (21). ChdC catalyzes the final step of heme biosynthesis, and therefore, the Δ*chdC* mutant lacks both peroxidase activity and endogenous heme (22). To distinguish which function is important for aerobic growth, we attempted to chemically complement growth of a Δ*chdC* mutant by supplementing it with exogenous heme. However, addition of exogenous heme is toxic unless bacterial cultures are pre-exposed to low levels of heme (23). Therefore, bacteria were first grown overnight in brain heart infusion (BHI), which contains heme, and then diluted into TSB containing or lacking heme (5 μM). Exogenous heme fully restored growth of a Δ*chdC* mutant to that of wt (Fig. 3D, dashed lines), suggesting that the aerobic growth defect of L. monocytogenes lacking *chdC* is primarily due to a lack of heme.

### Peroxidases important for acute peroxide toxicity.

**FIG 4:**
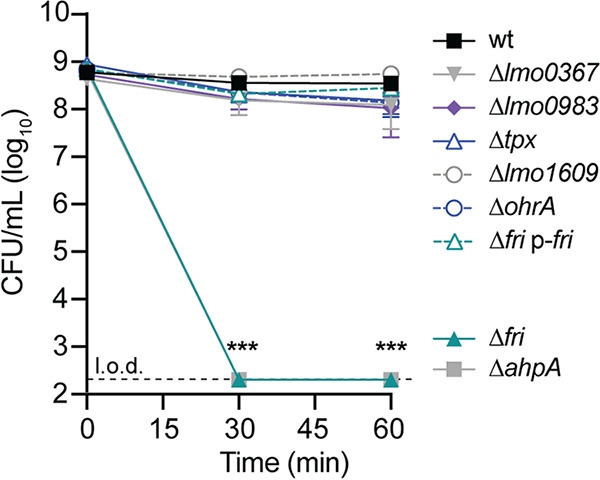
Acute peroxide toxicity. Bacteria were grown aerobically to mid-log phase before hydrogen peroxide (120 mM) was added. The dotted line indicates the limit of detection (l.o.d.). Data are means and SEM for four biological replicates. *P* values were calculated using a heteroscedastic Student's *t* test comparing each mutant to the wt. ***, *P* < 0.001.

We next investigated which peroxidases are required for detoxifying acute peroxide stress. Bacteria were grown overnight aerobically, as L. monocytogenes does not produce detectable peroxidase activity after anaerobic culture (12). Accordingly, only the mutants which replicated aerobically could be tested in this experiment. Each strain was grown to early logarithmic phase (optical density at 600 nm [OD_600_] = 0.6) before hydrogen peroxide (120 mM) was added for 1 h. At this concentration of peroxide, the wt decreased 1.7-fold over 1 h and most mutants exhibited similar resistance (Fig. 4). In contrast, both Δ*fri* and Δ*ahpA* mutants were rapidly killed, and no bacteria were detected at 30 or 60 min. These results are consistent with published reports demonstrating that *fri* and *ahpA* are important for survival in the presence of peroxide (14, 24, 25) and reveal that none of the other peroxidases tested provide nonredundant protection from peroxide under these conditions.

### Intracellular growth of peroxidase mutants.

**FIG 5:**
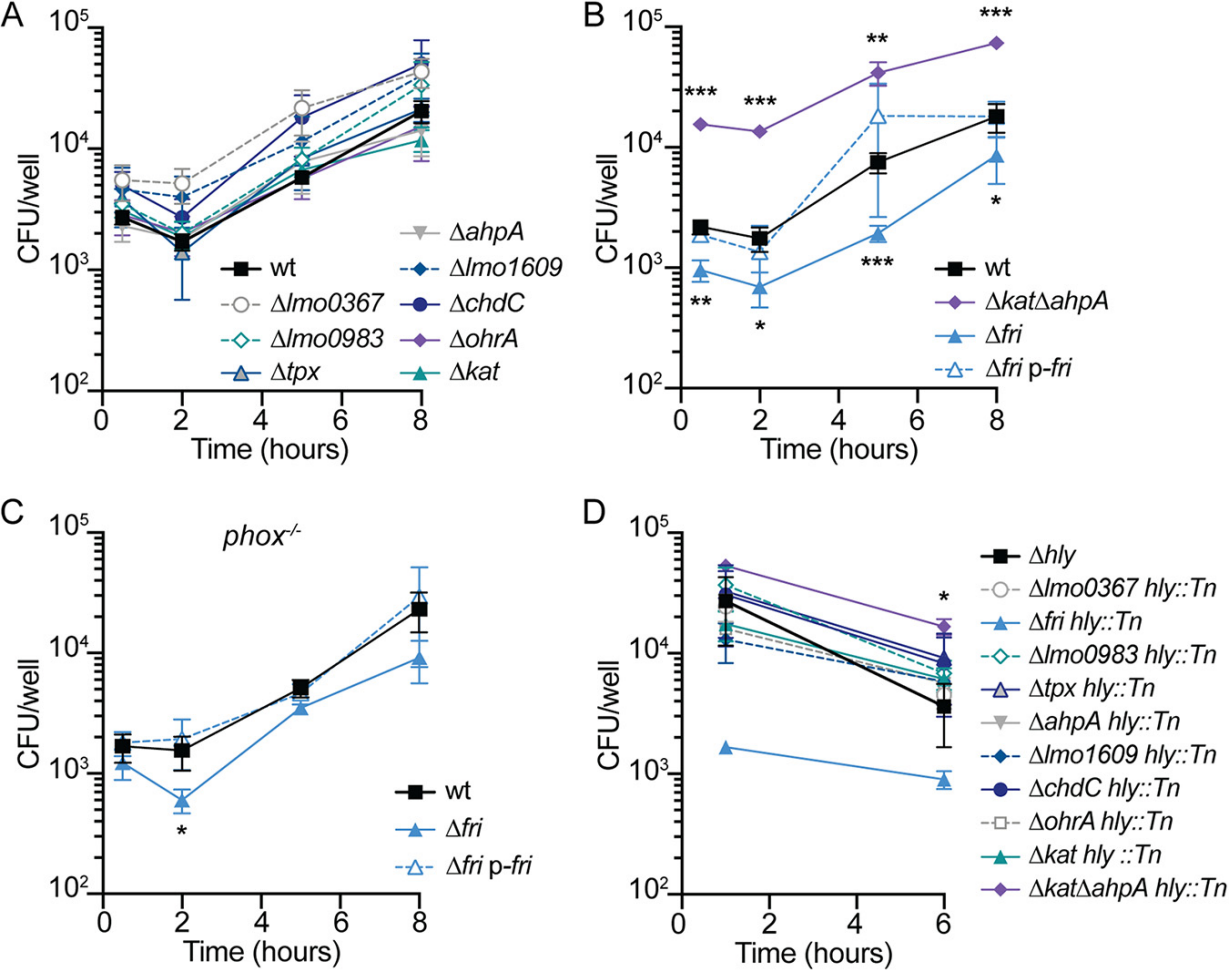
Intracellular survival and growth of peroxidase mutants in IFN-γ-activated BMDMs. (A) Intracellular growth curves of mutant strains that replicated at the same rate as the wt (*P* > 0.05 at each time point). (B) Intracellular growth curves in activated BMDMs. (C) Intracellular growth curves in activated *phox*^−/−^ BMDMs, which lack NADPH oxidase. (D) Survival of *hly* mutants trapped in the vacuoles of activated BMDMs. Although the error bars are too small to be visible, the Δ*fri hly*::Tn strain is not significantly different from the wt at 1 h (*P = *0.06). In all panels, data are means and SEM from at least three independent experiments. *P* values were calculated using a heteroscedastic Student's *t* test comparing each mutant to the wt grown under the same conditions. *, *P* < 0.05; **, *P* < 0.01; ***, *P* < 0.001.

During infection of macrophages, L. monocytogenes resides briefly in the phagosome, where it is bombarded by hydrogen peroxide produced by the host respiratory burst (1). Previous work demonstrated that Kat-mediated peroxide detoxification is not required for intracellular survival or growth in macrophages (12). To test which peroxidases are important intracellularly, we infected bone marrow-derived macrophages (BMDMs) with each strain at a multiplicity of infection (MOI) of 0.1 and enumerated CFU over time by plating anaerobically. All the mutants replicated similarly to the wt in resting BMDMs (data not shown). To ensure a robust innate immune response, we next infected BMDMs that had been pretreated with interferon gamma (IFN-γ) to activate the host respiratory burst (26). In these experiments, the majority of peroxidase mutants grew similarly to the wt (Fig. 5A). Surprisingly, the Δ*kat ΔahpA* double mutant exhibited a significant 5- to 8-fold increase in CFU at each time point compared to the wt (Fig. 5B). In contrast, the *Δfri* strain was significantly attenuated for intracellular replication in activated BMDMs, and this defect was genetically complemented by providing *fri* in *trans* (Fig. 5B).

Due to the role of Fri in iron storage, we sought to assess whether the growth defect of the Δ*fri* mutant was due to a lack of iron scavenging or peroxidase protection. To that end, intracellular growth curves were performed in gp91*^phox^*^−/−^ (*phox*^−/−^) macrophages, which lack the NADPH oxidase responsible for the host oxidative bursts and thus mimic the most common genetic defect observed in humans with CGD (3). In these immunodeficient cells, the Δ*fri* mutant exhibited a significant defect 2 h postinfection, but the intracellular bacterial burden was similar to that of the wt and the complemented strain at every other time point (Fig. 5C). Taken together, these results demonstrate that while the Δ*kat ΔahpA* mutant has an unexpected advantage during infection, *fri* is required for efficient infection of activated macrophages and this is at least partially alleviated in the absence of the host respiratory burst.

One hypothesis to explain the dispensability of the majority of peroxidases for intracellular growth (Fig. 5A) is that the host respiratory burst is ineffective against L. monocytogenes because the secreted pore-forming toxin listeriolysin O (LLO) allows rapid escape from the vacuole. To examine which peroxidases may be important specifically in the vacuolar environment, the gene encoding LLO (*hly*) was disrupted in each mutant background, and survival in the vacuole of IFN-γ-activated BMDMs was evaluated over time. In agreement with the intracellular growth curves, the majority of peroxidase mutants survived in the vacuole for 6 h at rates similar to that of the wt (Fig. 5D). The exceptions were the Δ*kat ΔahpA* mutant, which survived significantly better than the wt, and the Δ*fri* mutant, which exhibited an ∼1-log decrease in CFU at the earliest time point, although this was not statistically significant (*P = *0.06). Together, these data demonstrate that the only peroxidase important for vacuolar survival and intracellular growth in activated BMDMs is that encoded by *fri*.

### Intercellular spread.

**FIG 6:**
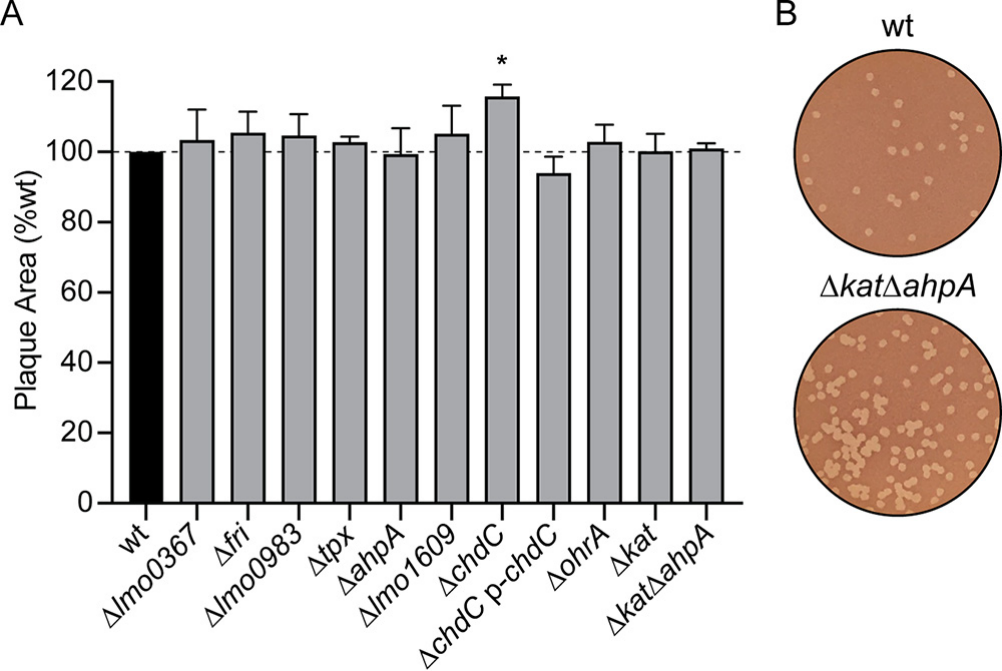
Intercellular spread of peroxidase mutants. (A) Plaque formation in L2 fibroblasts was evaluated for each strain. Data are means and SEM. The dashed line signifies the 100% level of the wt. (B) Representative images of plaques demonstrating the greater number of plaques formed by the Δ*kat ΔahpA* mutant than the wt.

We next sought to test the role of each peroxidase in virulence using a plaque assay, which is a measure of intracellular growth and cell-to-cell spread that is highly correlated with virulence in a murine model of infection (16, 27). In this assay, a monolayer of L2 murine fibroblasts is infected and immobilized in agarose containing gentamicin to kill extracellular bacteria. Three days postinfection, the living cells are stained with neutral red and the area of the plaques formed by L. monocytogenes are analyzed as a measure of intercellular spread. In this assay, all of the mutant strains formed plaques similar in size to or larger than those formed by the wt (Fig. 6A). Although the Δ*kat ΔahpA* double mutant formed plaques similar in size to those of the wt, the mutant exhibited a dramatic advantage at invading host cells. The Δ*kat ΔahpA* mutant formed approximately 5 times more plaques than wt (Fig. 6B). This is consistent with the increased bacterial burden observed in IFN-γ-activated BMDMs at the earliest time points (Fig. 5B) and suggests that the Δ*kat ΔahpA* mutant is better able to invade host cells.

## DISCUSSION

Bacterial pathogens must detoxify endogenously produced ROS and exogenous sources of oxidative stress during infection. Hydrogen peroxide is particularly dangerous, as it is an uncharged molecule that can penetrate membranes (5). In this study, we performed the first comparison of the expression and essentiality of all proteins with predicted peroxidase activity in L. monocytogenes. Importantly, of the nine peroxidases examined here, only ChdC has been biochemically characterized and shown to have peroxidase activity *in vitro* (22). Our results revealed that *kat* and *chdC* are required for aerobic growth in rich medium, while *ahpA* and *fri* are required to detoxify acute peroxide stress *in vitro*. The strain lacking both *kat* and *ahpA* exhibited the most severe aerobic growth defect but had a surprising advantage invading and surviving in host cells. While *fri*, *ahpA*, and *kat* were expressed during intracellular growth in macrophages, only *fri* was required for survival in IFN-γ-activated BMDMs. Considering that the host respiratory burst assaults invading bacteria with up to 100 μM peroxide in the phagosome (7), our results suggest a high degree of redundancy in the L. monocytogenes peroxide stress response.

Transcript analysis during aerobic growth revealed that L. monocytogenes expression of peroxidase-encoding genes was not dramatically altered in the *kat*-deficient strain compared to the wt. This was unexpected, as B. subtilis strains lacking *katA* or *ahpC* experience peroxide stress during aerobic growth that induces the PerR (peroxide stress response) regulon (28, 29). Similarly, the E. coli OxyR regulon is activated by the peroxide that accumulates when Hpx^−^ cells (Δ*katE* Δ*katG* Δ*ahpCF*) are grown aerobically (7, 30). Thus, one interpretation of our results is that L. monocytogenes *Δkat* succumbs to peroxide-mediated toxicity before the PerR regulon can be effectively activated (8). However, the Δ*kat ΔahpA* double mutant is even more susceptible to endogenous redox stress *in vitro* but undergoes undefined regulatory changes that result in increased invasion, survival, and replication in activated macrophages. Ongoing research is aimed at deciphering the regulatory changes that occur in the absence of *kat* and *ahpA* that lead to increased virulence.

Lmo1604 is annotated as an alkyl hydroperoxide reductase (Ahp) based on similarity to other enzymes. The classical Ahp system is composed of two components: the peroxiredoxin (AhpC) and a dedicated reductase (AhpF) that reduces and recycles AhpC and is typically encoded by a sequence adjacent to *ahpC* (31). Many organisms encode multiple Ahp proteins; however, L. monocytogenes encodes only Lmo1604, and there is no adjacent reductase. L. monocytogenes Lmo1604 shares 75% and 33% identity with B. subtilis AhpA and AhpC, respectively. While two previous publications referred to this protein as Prx (24, 25), herein we refer to Lmo1604 as AhpA to reflect its similarity to B. subtilis AhpA and the fact that it is a noncanonical Ahp that lacks a dedicated reductase. We found that *ahpA* is expressed during intracellular replication but is not required for growth in IFN-γ-activated macrophages or prolonged survival in the vacuole. These results are consistent with published work showing that the Δ*ahpA* mutant had no defect in macrophages or during infection of mice (25). However, *ahpA* was required for surviving acute peroxide stress *in vitro* and was important for detoxifying endogenous peroxide during aerobic replication in the absence of catalase. Together, these results suggest a role for L. monocytogenes AhpA in peroxide detoxification that is masked in wt cells by the activity of catalase. Interestingly, the opposite is true in E. coli, where Ahp is the primary scavenger of endogenous peroxide and catalase is important only in the presence of high concentrations of peroxide (30).

Hydrogen peroxide toxicity and iron are inextricably linked due to the Fenton reaction, in which ferrous iron reacts with hydrogen peroxide to generate hydroxyl radicals that damage DNA (7). Unsurprisingly, several of the putative peroxidases in this study are also involved in maintaining iron homeostasis in the cell, including the heme-dependent peroxidase Lmo0367, the bacterial ferritin Fri, the terminal heme biosynthesis enzyme ChdC, and the heme-dependent catalase Kat. Additional studies are necessary to assess the roles of these enzymes in iron influx, oxidation, and storage in response to oxidative stress.

One method used by bacteria to mitigate the danger of free iron and the Fenton reaction is to sequester iron within bacterial ferritin proteins, which are multimeric protein shells that can store up to 4,500 iron atoms. The amino acid sequence of L. monocytogenes Fri is identical to that of the Listeria innocua Dps protein, which has been biochemically characterized as a bacterial ferritin. *L. innocua* Dps was named due to its structural similarity to Dps family proteins (DNA-binding protein from starved cells), although it does not bind DNA (32). *L. innocua* Dps has a smaller internal diameter than typical ferritins and therefore can store only ∼400 iron atoms per shell. The protection afforded by *L. innocua* Dps is due to the hydrogen peroxide-mediated iron oxidation, which occurs rapidly and in a manner that prevents Fenton chemistry (32, 33). Based on the identity of the proteins, we predict that L. monocytogenes Fri has a similar function and uses peroxide to oxidize and store iron. It is therefore not possible to distinguish between the importance of the peroxidase activity of Fri and its role in iron homeostasis in the cell.

The role of Fri in L. monocytogenes has been investigated previously by several groups, and in fact, the protein encoded by *lmo0943* has been given multiple different names, including Flp (ferritin-like protein) (34), Frm (ferritin-like protein from L. monocytogenes) (35), and Frl (ferritin-like protein in *Listeria* species) (36). Here, we kept the more common name Fri (14, 15, 37). Our results are consistent with the literature showing that *fri* expression increases 8-fold upon entry into stationary phase and a Δ*fri* mutant is more sensitive to peroxide stress when treated in log phase (14, 37). In addition, we identified a critical role for Fri in the transition from anaerobiosis to aerobic replication. We also determined that *fri* expression is increased during intracellular growth and accordingly, *fri* is required for growth in activated BMDMs. This requirement was partially alleviated in host cells incapable of mounting an effective respiratory burst, suggesting that host-derived peroxide contributes to limiting intracellular replication of the Δ*fri* mutant.

L. monocytogenes and Staphylococcus aureus mutants deficient in heme biosynthesis enzymes form small colonies on solid media and grow poorly in aerobic liquid cultures (12, 38). While *chdC* was previously reported to be essential (21), we generated a Δ*chdC* mutant in anaerobic growth conditions and observed that it was indeed unable to replicate aerobically in the absence of exogenous heme. This severe growth defect resembled that of L. monocytogenes Δ*hemEH*, which also cannot produce heme (12). As our data attribute the Δ*chdC* growth defect to the heme deficiency, it is unlikely that ChdC peroxidase activity plays a primary role in endogenous peroxide detoxification. However, our results raise an interesting question: why is heme biosynthesis required for L. monocytogenes aerobic growth? Heme is an essential cofactor for cytochrome oxidases of the electron transport chain, and therefore, S. aureus *hem* mutants are impaired for growth due to a lack of aerobic respiration (38, 39). However, L. monocytogenes lacking one or both terminal cytochrome oxidases are only moderately impaired for aerobic growth, compared to the complete lack of replication observed for Δ*chdC* and Δ*hemEH* mutants (12, 40). Interestingly, S. aureus requires heme biosynthesis for virulence in murine models of acute infection (41). In contrast, L. monocytogenes mutants lacking *hemEH* or *chdC* are not defective for intracellular replication and, in fact, exhibit increased intercellular spread compared to the wt (12; N. H. Schwardt, unpublished observations). These results suggest either that heme biosynthesis is dispensable for L. monocytogenes pathogenesis or that exogenous host-derived heme can support growth of the Δ*chdC* mutant during infection. Ongoing research aims to determine the role of heme in L. monocytogenes aerobic growth and virulence.

In our assays, we did not observe phenotypes for L. monocytogenes lacking *lmo0367*, *lmo0983*, *tpx*, *lmo1609*, or *ohrA*, and little is known about their functions. Lmo0367 shares 52% amino acid identity with the B. subtilis YwbN/EfeB protein, a DyP-type peroxidase that is transported as a folded protein across the cytoplasmic membrane via the twin-arginine translocation (Tat) pathway (42, 43). While the protein localization and function of L. monocytogenes Lmo0367 have not been examined, the corresponding gene was found to be regulated by the ferric uptake regulator Fur and expression was consequently decreased in response to heme stress (44, 45). A screen for L. monocytogenes genes important for osmotic stress and desiccation identified a transposon in *lmo0983*, encoding a putative glutathione peroxidase, although this mutant was not further characterized (46). Tpx and Lmo1609 share 63% and 58% amino acid identity with their respective homologues in B. subtilis, and both are activated by Spx in that organism, supporting their role in the oxidative stress response (47). The L. monocytogenes *ohrA* mutant was previously found to be attenuated in a plaque assay and for intracellular replication in BMDMs (16). In this study, the Δ*ohrA* strain was not attenuated, and we hypothesize this discrepancy is due to the fact that herein, the bacteria were grown overnight anaerobically before infecting cells, whereas previous experiments grew bacteria aerobically. Future research will elucidate the biochemical functions of these understudied peroxidases in L. monocytogenes and other *Firmicutes* and the role of these peroxidases during aerobic growth and intracellular infection.

Oxidative stress is abundant in the environment during aerobic growth and during infection. In this work, we focused on peroxide stress, although superoxide is also generated endogenously and encountered exogenously in host phagocytes (1). L. monocytogenes produces a single manganese-dependent superoxide dismutase (MnSOD) that is required for infection (48, 49). MnSOD converts the superoxide produced by NADPH oxidase to hydrogen peroxide, which then needs to be detoxified by catalases and peroxidases. Our results demonstrate that the majority of peroxidases are individually dispensable for infection of mammalian cells and suggest redundancy in these antioxidants. Identifying expression changes in the Δ*kat ΔahpA* strain will reveal the compensatory factors allowing this double mutant to more efficiently infect macrophages, despite the *in vitro* sensitivity of this strain to peroxide. Future investigations will build on the results described herein to provide further insight into the L. monocytogenes peroxide detoxification strategy during infection.

## MATERIALS AND METHODS

### Ethics statement.

This study was carried out in strict accordance with the recommendations in the *Guide for the Care and Use of Laboratory Animals* of the National Institutes of Health (50). All protocols were reviewed and approved by the Institutional Animal Care and Use Committee at the University of Washington (protocol 4410-01).

### Bacterial strains and culture conditions.

L. monocytogenes strains were derived from the wt strain 10403S and are listed in Table 2. E. coli strains are listed in Table 3. L. monocytogenes was cultured in either tryptic soy broth (TSB) or brain heart infusion (BHI), aerobically in the dark, with shaking at 37°C unless other conditions are indicated. Anaerobic conditions were established by growing bacteria in closed containers with GasPak EZ Anaerobe gas-generating pouches (Becton Dickinson) or placing cultures in degassed medium inside a closed-system anaerobic chamber (Don Whitley Scientific A35 anaerobic work station). Unless otherwise stated, the chemicals used were purchased from Sigma-Aldrich. The following concentrations of antibiotics were used: streptomycin, 200 μg ml^−1^; chloramphenicol, 10 μg ml^−1^ (E. coli) and 7.5 μg ml^−1^ (L. monocytogenes); carbenicillin, 100 μg ml^−1^; erythromycin, 1 μg ml^−1^; and tetracycline, 1 μg ml^−1^.

**TABLE 2:** L. monocytogenes strains used in this study

Strain	Description	Reference(s)
MLR-L001	10403S	55, 56
MLR-L948	Δ*lmo0367*	This work
MLR-L960	Δ*fri* (*lmo0943*)	This work
MLR-L954	Δ*lmo0983*	This work
MLR-L959	Δ*tpx* (*lmo1583*)	This work
MLR-L879	Δ*ahpA* (*lmo1604*)	This work
MLR-L953	Δ*lmo1609*	This work
MLR-L944	Δ*chdC* (*lmo2113*)	This work
MLR-L174	Δ*ohrA* (*lmo2199*)	16
MLR-L828	Δ*kat* (*lmo2785*)	12
MLR-L880	Δ*kat* Δ*ahpA*	This work
MLR-L971	Δ*fri* p-*fri*	This work
MLR-L850	pPL1.p*Hyper*-*gfp* (pH-*gfp*)	This work
MLR-L984	pH-*gfp pHyper*-*rfp*	This work
MLR-L985	pH-*gfp P-lmo0367*-*rfp*	This work
MLR-L986	pH-*gfp P-fri*-*rfp*	This work
MLR-L987	pH-*gfp P-lmo0983*-*rfp*	This work
MLR-L988	pH-*gfp P-tpx*-*rfp*	This work
MLR-L983	pH-*gfp P-ahpA*-*rfp*	This work
MLR-L989	pH-*gfp P-lmo1609*-*rfp*	This work
MLR-L982	pH-*gfp P-chdC*-*rfp*	This work
MLR-L990	pH-*gfp P-ohrA*-*rfp*	This work
MLR-L981	pH-*gfp P-kat*-*rfp*	This work
MLR-L991	*hly*::*himar1* (Tn)	10
MLR-L992	Δ*lmo0367 hly*::Tn	This work
MLR-L1000	Δ*fri hly*::Tn	This work
MLR-L995	Δ*lmo0983 hly*::Tn	This work
MLR-L996	Δ*tpx hly*::Tn	This work
MLR-L999	Δ*ahpA hly*::Tn	This work
MLR-L997	Δ*lmo1609 hly*::Tn	This work
MLR-L994	Δ*chdC hly*::Tn	This work
MLR-L998	Δ*ohrA hly*::Tn	This work
MLR-L993	Δ*kat hly*::Tn	This work
MLR-L1001	Δ*kat* Δ*ahpA hly*::Tn	This work

**TABLE 3:** E. coli SM10 strains used in this study

Strain	Description	Reference
SM10	For transconjugation	57
MLR-E975	pPL2t.P-*lmo0367*-*rfp*	This work
MLR-E978	pPL2t.P-*fri*-*rfp*	This work
MLR-E976	pPL2t.P-*lmo0983*-*rfp*	This work
MLR-E977	pPL2t.P-*tpx*-*rfp*	This work
MLR-E974	pPL2t.P-*ahpA*-*rfp*	This work
MLR-E979	pPL2t.P-*lmo1609*-*rfp*	This work
MLR-E973	pPL2t.P-*chdC*-*rfp*	This work
MLR-E980	pPL2t.P-*ohrA*-*rfp*	This work
MLR-E972	pPL2t.P-*kat-rfp*	This work
MLR-E970	pPL2.*fri*	This work

Tissue culture cells were routinely cultured in high-glucose Dulbecco modified Eagle medium (DMEM) at 37°C with 5.5% CO_2_. L2 fibroblasts were generated previously from L929 cells (16, 27). L2s and J774 macrophages were maintained in medium containing 10% fetal bovine serum (FBS) (HyClone), 2 mM l-glutamine (Gibco), and 1 mM sodium pyruvate (Gibco). Bone marrow-derived macrophages (BMDMs) were derived as previously described (16, 51). Bone marrow was from C57BL/6 mice purchased from The Jackson Laboratory or from B6.129S-*Cybb^tm1Din^* (also known as gp91*^phox^*^−/−^) mice, a generous gift from the Fang laboratory (University of Washington) and originally from The Jackson Laboratory. BMDMs were cultured in 20% FBS, 2 mM l-glutamine, 1 mM sodium pyruvate, β-mercaptoethanol (BME; 55 μM), and 10% macrophage colony-stimulating factor (M-CSF) (conditioned medium from 3T3 cells expressing M-CSF).

### Cloning and plasmid construction.

In-frame deletions were carried out via the conjugatable suicide vector pLIM1 and allelic exchange (plasmid provided as a generous gift from Arne Rietsch, Case Western Reserve University). Complementation of in-frame deletions at ectopic loci were accomplished using pPL2 integration plasmids as previously described (20). Whole genes accompanied by promoter regions were amplified and ligated into pPL2. Constructs were transformed into E. coli SM10 cells and introduced into L. monocytogenes mutants via transconjugation. Complemented mutants were confirmed by antibiotic resistance and Sanger sequencing.

Fluorescent transcriptional reporters were engineered to express *gfp* constitutively from the HyPer promoter using the integrative plasmid pPL1 (16, 20). This was integrated into the chromosome of a phage-cured wt L. monocytogenes strain DP-L4056 (20). Next, the promoter regions of peroxidase genes were amplified and ligated to mTAG-RFP via NEBuilder HiFi DNA assembly. The mTAG-RFP was amplified from DP-L6508 (16). The promoter-*rfp* fusions were ligated into pPL2t and confirmed via PCR and Sanger sequencing. E. coli SM10 harboring the pPL2t.Promoter-*rfp* constructs were mated with MLR-L850 (pPL1.p*Hyper*-*gfp*) to generate the two-color transcriptional reporter strains. Integration was confirmed by antibiotic resistance and PCR.

Peroxidase mutants unable to escape from the vacuole were generated by transducing each mutant strain with a phage lysate produced in the *hly*::*himar1* background, as previously described (16). Briefly, U153 phage were mixed with the appropriate donor strain and incubated at 30°C in LB soft agar overnight. Phage lysates were eluted from agar, filter sterilized, and added to recipient strains for 30 min at room temperature. Transductions were plated on antibiotic containing agar and incubated at 37°C. Insertions in *hly* were confirmed by PCR.

### Growth curves and peroxide toxicity.

For anaerobic growth, colonies were inoculated into TSB and incubated at 37°C in closed containers containing anaerobic gas-generating pouches (GasPak EZ; BD). Anaerobic overnight cultures were normalized to an OD_600_ of 0.02 in 25 ml of TSB in 250-ml flasks and grown aerobically with shaking at 37°C. At each time point, bacteria were serially diluted, plated on BHI agar, and grown anaerobically to enumerate CFU.

To assess the response to acute peroxide toxicity, bacteria grown overnight in TSB at 37°C with shaking were back-diluted to an OD_600_ of 0.1 and incubated for 2 h to an OD_600_ of ∼0.6. Hydrogen peroxide (120 mM; Sigma) was added, and cultures were incubated with shaking for 1 h. At each time point, bacterial cultures were serially diluted and plated on BHI to enumerate CFU.

### Quantitative RT-PCR of bacterial transcripts.

Bacteria grown anaerobically overnight in degassed TSB were normalized to an OD_600_ of 0.02 in 50 ml medium in 500-ml flasks and incubated at 37°C in the dark, with shaking. At each time point, 5 to 7 ml of bacterial culture was removed, mixed with ice-cold methanol (1:1), pelleted by centrifugation, flash frozen, and stored at −80°C. Nucleic acids were harvested as previously described using acid phenol-chloroform extraction and bead beating (16). RNA was precipitated overnight and washed in ethanol, and RT-PCR was performed with iScript reverse transcriptase (Bio-Rad). Quantitative PCR was performed with iTaq Universal SYBR green Supermix (Bio-Rad) according to manufacturers’ recommendations. Transcripts were normalized to that of 16S rRNA and fold change was calculated using the comparative cycle threshold (*C_T_*) method.

### Macrophage growth curves.

BMDMs were seeded at a concentration of 6 × 10^5^ cells per well in tissue culture (TC)-treated 24-well plates the day before infection. BMDMs were activated by incubating the monolayer with recombinant murine IFN-γ (100 ng/ml; PeproTech) overnight and during infection. Overnight bacterial cultures were grown anaerobically in TSB (the Δ*fri* mutant aerobically) at 30°C statically, washed twice with PBS, and resuspended in warmed BMDM medium (16). BMDMs were infected at an MOI of 0.1 for 30 min before cells were washed twice with PBS, and then BMDM medium containing gentamicin (50 μg/ml) was added to each well. To measure bacterial growth, cells were washed twice with PBS and then lysed by incubating with 250 μl cold PBS with 0.1% Triton X-100 for 5 min at room temperature, followed by serial dilutions and plating on BHI agar to enumerate CFU. For the *phox*^−/−^ growth curves, all strains were incubated aerobically in TSB.

### Infections and flow cytometry.

J774 cells were plated in 12-well TC-treated dishes at 10^6^ cells per well. Generally, L. monocytogenes transcriptional reporter strains were grown to mid-log phase at 37°C with shaking. After being washed twice and resuspended in phosphate-buffered saline (PBS), bacterial suspensions were added to the cells at an MOI of 10. At 1 h postinfection, cells were washed twice with PBS, and medium containing gentamicin (50 μg/ml) was added to each well. At 6 h postinfection, the cells were washed twice with PBS, treated with 0.25% trypsin (Gibco), and resuspended in an equal volume of medium. The cells were then fixed with 2% formaldehyde, washed twice with flow buffer (PBS containing 5% FBS), and resuspended in 300 μl flow buffer. Flow cytometry was performed on an LSR II flow cytometer (BD) and analyzed using FlowJo (FlowJo, LLC). Cells were discriminated from debris by forward scatter area (FSC-A) and side scatter area (SSC-A). Single cells were gated using FSC-A and forward scatter height (FSC-H). The GFP gate was set to include 5% of the uninfected sample; this gate represents infected cells and is referred to as GFP positive. Within the infected-cell population, the RFP gate was set to include 5% of cells infected with pH-gfp; this gate is referred to as RFP positive. Reported values represent percent RFP-positive cells within GFP-positive cells of a given sample.

### Plaque assays.

Plaque assays were performed as previously described (16, 27, 52, 53). Briefly, TC-treated 6-well dishes were seeded with 1.2 × 10^6^ L2 murine fibroblasts per well. L. monocytogenes strains were incubated in BHI overnight at 30°C, static. Overnight cultures were diluted 1:10 in sterile PBS, and 5 to 10 μl was used to infect each well. One hour postinfection, cells were washed twice with PBS and 3 ml of molten agarose-DMEM solution was added to each well. This solution consisted of a 1:1 mixture of 2× DMEM (Gibco) and 1.4% SuperPure agarose LE (U.S. Biotech Sources, LLC) containing gentamicin (10 μg/ml). Three days postinfection, 2 ml of molten agarose-DMEM solution containing neutral red was added to each well to visualize plaques. After 24 h, the plates were scanned, and the plaque areas measured using ImageJ software (54). The area of at least 20 plaques was measured for each strain and normalized to that of the wt.
